# Ultrasound-Assisted One-Pot Cloud Point Extraction for Iron Determination Using Natural Chelating Ligands from *Dipterocarpus intricatus* Dyer Fruit

**DOI:** 10.3390/molecules27175697

**Published:** 2022-09-04

**Authors:** Sam-ang Supharoek, Bordin Weerasuk, Watsaka Siriangkhawut, Kate Grudpan, Kraingkrai Ponhong

**Affiliations:** 1 Department of Medical Science, Amnatcharoen Campus, Mahidol University, Amnat Charoen 37000, Thailand; 2 Department of Chemistry and Center for Innovation in Chemistry, Faculty of Science, Mahidol University, Bangkok 10400, Thailand; 3 Creative Chemistry and Innovation Research Unit, Department of Chemistry and Center of Excellence for Innovation in Chemistry, Faculty of Science, Mahasarakham University, Maha Sarakham 44150, Thailand; 4 Department of Chemistry, Faculty of Science and Center of Excellence for Innovation in Analytical Science and Technology for Biodiversity-based Economic and Society, Chiang Mai University, Chiang Mai 50200, Thailand; 5 Multidisciplinary Research Unit of Pure and Applied Chemistry (MRUPAC), Department of Chemistry and Center of Excellent for Innovation in Chemistry, Faculty of Science, Mahasarakham University, Maha Sarakham 44150, Thailand

**Keywords:** ultrasound-assisted one-pot cloud point extraction, *Dipterocarpus intricatus* Dyer, green analytical method, iron determination, natural reagent, spectrophotometry

## Abstract

An ultrasound-assisted, one-pot cloud point extraction was developed for the determination of iron in vegetable samples by UV-Visible spectrophotometry. This method was based on the complexation of iron with an environmentally-friendly natural chelating agent extracted from *Dipterocarpus intricatus* Dyer fruit at pH 5.5 in the presence of Triton X-114. Reagent extraction, complexation, and preconcentration were performed simultaneously using ultrasound-assisted extraction at 45 °C. The surfactant-rich phase was diluted with ethanol and loaded through a syringe barrel packed with cotton that acted as a filter to trap the reagent powder. Analyte-entrapped on cotton was eluted using 0.1 mol·L^−1^ nitric acid solution. Filtrate and eluate solutions were measured absorbance of the dark-blue product at 575 nm. Influential parameters for the procedure were investigated. Under the optimum experimental conditions, the calibration curve was linear, ranging from 0.1 to 1.0 mg·L^−1^ with *r*^2^ = 0.997. Limits of detection and quantification were 0.03 and 0.09 mg·L^−1^, respectively while precision values of intra-day and inter-day were less than 5%. Recovery at 0.5 mg·L^−1^ ranged from 89.0 to 99.8%, while iron content in vegetable samples ranged from 2.45 to 13.36 mg/100 g. This method was cost-effective, reliable, eco-friendly, and convenient as a green analytical approach to determining iron content.

## 1. Introduction

Iron is the most abundant essential trace element in the human body. Iron is necessary for the synthesis of hemoprotein that functions as oxygen transport and oxidative metabolism. The median dietary intake of iron is 16 to 18 mg/day for men and 12 mg/day for women [1]. Iron occurs as a natural element of all foods of plant and animal origin and may also be present in drinking water. Vegetables are important for human health as sources of various antioxidant vitamins (vitamin A, vitamin C, and vitamin E), minerals (iron and calcium), phytochemical compounds, and dietary fiber.

UV-Visible spectrophotometry based on commercially available synthetic reagents such as 2-(2,3-dihydroxy-4-oxocyclobut-2-enylidene) hydrozinecarbothiamide [2], thiocyanate [3], 2,2′-azino-bis(3-ethylbenzothiazoline-6-sulfonate) (ABTS) [4], 1, 10-phenanthroline [5,6], 2-(5-bromo-2-pyridylazo)-5-(diethylamino)-phenol (Br-PADAP) [7], desferrioxamine B [8], and 3-hydroxy-4-pyridinone [9] has been developed for the determination of iron. These reagents have many disadvantages. They are expensive and generate large amounts of hazardous chemical waste that is harmful to human health and causes environmental pollution. Recently, improving the existing methods through the development of novel approaches such as green chemistry has attracted increased attention.

The concept of green analytical chemistry (GAC) was introduced in 1995 as a clean, environmentally-friendly analytical method. GAC has now been widely accepted by researchers and technicians as a pragmatic approach to reducing expenses while maintaining environmental sustainability. GAC has been implemented in many fields including spectroscopy (atomic and molecular) and electroanalytical and separation methods covering chromatography and electrophoresis techniques [10,11].

In molecular spectroscopic fields especially UV-Visible spectrophotometry, many publications have reported natural plant extractions as alternatives to commercially available reagents for the development of environmentally-friendly analytical methods, such as pumpkin for benzoyl peroxide assay [12], turmeric (*Curcuma longa* L.) for acetic acid assay [13], Indian mulberry root [14], heartwood of sappan wood [15] and Indian almond leaves [16] for aluminum assay, slippery elm (*Ulmus rubra*) leaf to determine trace amounts of molybdenum(VI) [17], and Oriental plane tree (*Platanus orientalis*) for the determination of zirconium [18]. Natural reagents have been developed as green analytical methods to quantify iron such as Indian gooseberry [19], guava leaves [20], green tea [21], betel nut [22,23], lead tree [24], and *Smilax china* root [25].

A cloud point extraction method was employed to increase sensitivity and eliminate background interference before analysis [26]. Cloud point extraction is widely used to enhance sensitivity using micelle-mediated surfactants in a separation and preconcentration procedure [26]. Many applications of micellar phase extraction including the determination of metal ions [27] based on the formation of a complex using thiazolylazo dyes [28], diethyldithiocarbamate [29,30], dithizones [31], 4-(*p*-chlorophenyl)-1-(pyridin-2-yl)thiosemicarbazide [32], and pyrrolidine dithiocarbamate [33] have been reported. Major advantages of this extraction method are simple experimental procedures, low cost, high preconcentration factors, and environmental safety in agreement with “green chemistry” principles [27]. 

Ultrasound-assisted extraction (UAE) was utilized to enhance extraction efficiency through the cavitation phenomenon. The implosion of cavitation bubbles increased pressure near the solid surface and allowed extraction solvents to penetrate solid matrices [34,35]. UAE technology reduced both time and temperature with a decrease in energy consumption and an increase in security, safety, and environmental friendliness compared with solvent extraction, mechanical expelling, supercritical extraction, and microwave extraction [36,37,38] for the extraction of thermolabile and bioactive compounds, while maintaining extract quality. UAE is a simple, rapid, and green extraction method for the recovery of polysaccharides [39] and phenolic compounds from *Cucurbita pepo* seed [40], *Eryngium caucasicum* leaves [41], and *Trigonella foenum-graecum* L. seed [42].

*Dipterocarpus intricatus* Dyer was introduced from Thailand [43] and is now widely distributed in tropical regions of Asia, particularly in Southeast Asia, such as Cambodia, Indonesia, Malaysia, Myanmar, and Vietnam. This deciduous species, 15–25 m tall, produces flowers and fruit during the summer. Eight phenolic constituents are found in *Dipterocarpus intricatus* Dyer stem including (Z)-ε-viniferin, (–)-4′-Omethylepigallocatechin 3-gallate, 11-Ogalloylbergenin, bergenin, 4-hydroxybenzal-dehyde, vanillin, vanillic acid, and syringic acid [44]. Tannic acid is a naturally occurring complex polyphenolic compound found in most aerial plant tissues [45]. Antioxidants have been studied in flowers of *Dipterocarpus intricatus* Dyer [46], with no reported phenolic compounds in *Dipterocarpus intricatus* Dyer fruit.

A simple aqueous extract of *Dipterocarpus intricatus* Dyer fruit, consisting of phenolic compounds as a natural chelation agent, was used for the determination of iron. No previous studies have reported using crude natural reagent extract from *Dipterocarpus intricatus* Dyer fruit as a chelating ligand to form a complex with iron coupling by micellar phase extraction. Crude reagent extract was compared with standard tannic acid to evaluate the chemical composition. Reagent extraction, complexation, and preconcentration require many types of glassware, with increased analysis time. Thus, to overcome these steps, a one-pot operation for reagent extraction, complexation, and preconcentration by cloud point extraction was developed for the determination of iron quantity in vegetables. 

## 2. Results and Discussion

### 2.1. Natural Reagent Ability to Associate with Metal Ions

**Figure 1:**
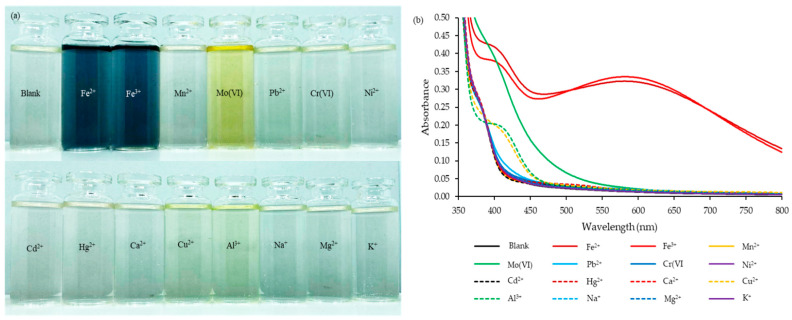
Results of the reaction between the natural reagent and various metal ions (**a**) Digital images; (**b**) absorption spectra under 1 mL of natural reagent, 10 mg·L^−1^ of each metal ion (**―** Blank; **―** Fe^2+^; **―** Fe^3+^; **―** Mn^2+^; **―** Mo(VI); **―** Pb^2+^; **―** Cr(VI); **―** Ni^2+^; **----** Cd^2+^; **----** Hg^2+^; **----** Ca^2+^; **----** Cu^2+^; **----** Al^3+^; **----** Na^+^; **----** Mg^2+^, and **----** K^+^) and 5.0 mL of 1.0 mol·L^−1^ acetate buffer pH 5.5.

The ability of the natural reagent to associate with metal ions such as Fe^2+^, Fe^3+^, Mn^2+^, Mo(VI), Pb^2+^, Cr(VI), Ni^2+^, Cd^2+^, Hg^2+^, Ca^2+^, Cu^2+^, Al^3+^, Na^+^, Mg^2+^, and K^+^ was investigated using 1.0 mL of natural reagent. A total of 1.0 mL of 100 mg·L^−1^ of each metal ion with final concentration was 10 mg·L^−1^ and 5.0 mL of 1.0 mol·L^−1^ acetate buffer pH 5.5. Digital image results are presented in Figure 1a. Only iron(III) and iron(II) ions were associated with the natural reagent, providing a dark-blue product that was maximally absorbed at a wavelength of 575 nm (Figure 1b). Absorbance values obtained by iron(III) with the natural reagent complex were slightly higher than iron(II). Therefore, this developed method quantified total iron in terms of the ferric (Fe^3+^) form.

**Figure 2:**
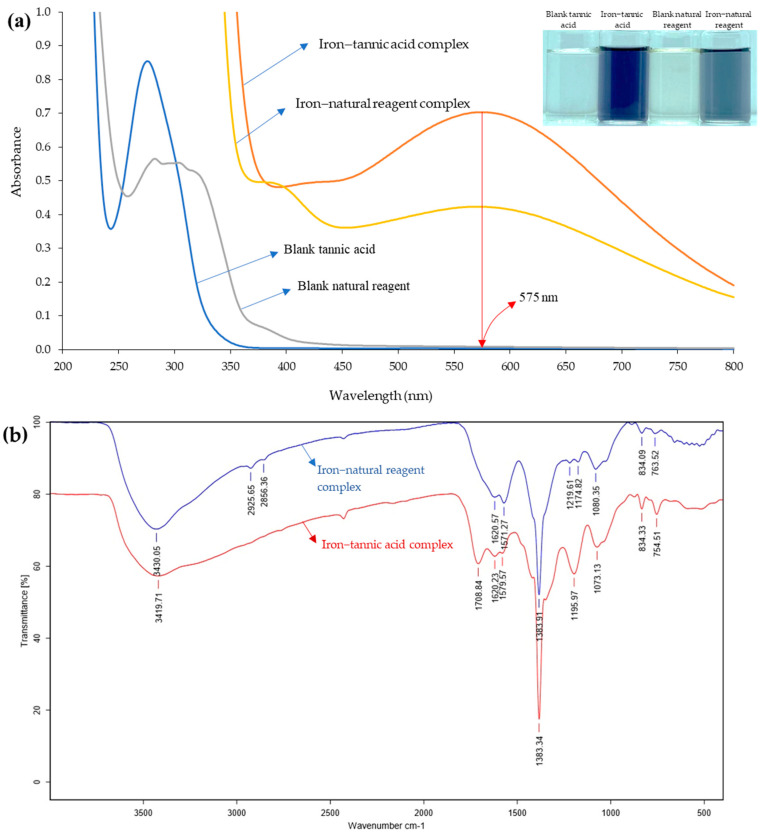
Results of natural reagent−iron complex and tannic−iron complex (**a**) Absorption spectra and digital images of iron associated with natural reagent and tannic acid and (**b**) FT−IR spectra of iron-natural reagent and iron−tannic acid complex.

Absorption spectra of the natural reagent extracted using deionized water and reaction with iron(III) are presented in Figure 2a. The spectrum of the natural reagent with iron(III) provided maximum absorption at 575 nm is the same as the spectrum obtained from the tannic acid reaction with iron(III). Furthermore, the IR spectrum of the natural reagent-iron(III) complex was similar to the IR spectrum of the tannic acid-iron(III) complex, as shown in Figure 2b. The characteristics of the IR spectrum for the natural reagent-complex showed a stretch of the -OH band at 3430.35 cm^−1^, (C-C) stretch and (C-H) deformation in the plane of the benzene ring at 1620.57 cm^−1^, (C-C) stretch and (C-H) deformation in the plane of the benzene ring, (C-O) stretch of phenolic at 1571.27 cm^−1^, (C-O) stretch and (O-H) deformation of phenolic, (C-C) stretch and (C-H) deformation in the plane of the benzene ring at 1383.91 cm^−1^, (O-H) deformation in the plane of phenolic and carboxylic acid, (C-C) stretch and (C-H) deformation in the plane of the benzene ring at 1174.82 cm^−1^, (C-O) stretch of phenolic at 1080.35 cm^−1^, (C-H) deformation out of the plane of the benzene ring at 834.01 cm^−1^, and (C-H) torsion of the benzene ring at 763.51 cm^−1^.

**Figure 3:**
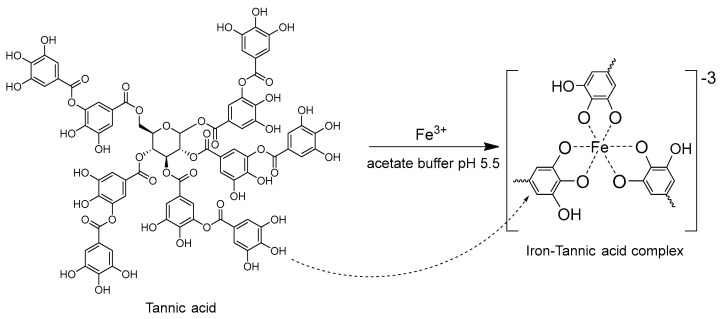
Complex formation between tannin and iron(III) [47,48].

Major chemical substances in *Dipterocarpus intricatus* Dyer fruit may include tannin or tannic acid, theogallin, gallic acid, and ellagic acid that act as metal chelators [47,48] in the reaction between iron(III) and tannic acid to provide the complex blue color under pH 5.5, as complex formation between iron and tannin as shown in Figure 3. 

### 2.2. Optimization of Ultrasound-Assisted One-Pot Cloud Point Extraction for Quantification of Iron

The developed method for the extraction of tannic acid from natural *Dipterocarpus intricatus* Dyer fruit was simultaneously operated with cloud point extraction of the iron-tannic complex under ultrasonication. The surfactant-rich phase was diluted and loaded into a syringe column packed with cotton before spectrophotometric determination. Various parameters affecting the proposed method including the amount of natural reagent, pH, buffer concentration, surfactant concentration, vortex time, incubation temperature, irradiation time, centrifugation speed and time, ethanol volume, and elution solvent were investigated using the univariate method at 1.0 mg·L^−1^ of iron(III) standard.

#### 2.2.1. Amount of *Dipterocarpus intricatus* Dyer Fruit Powder

**Figure 4:**
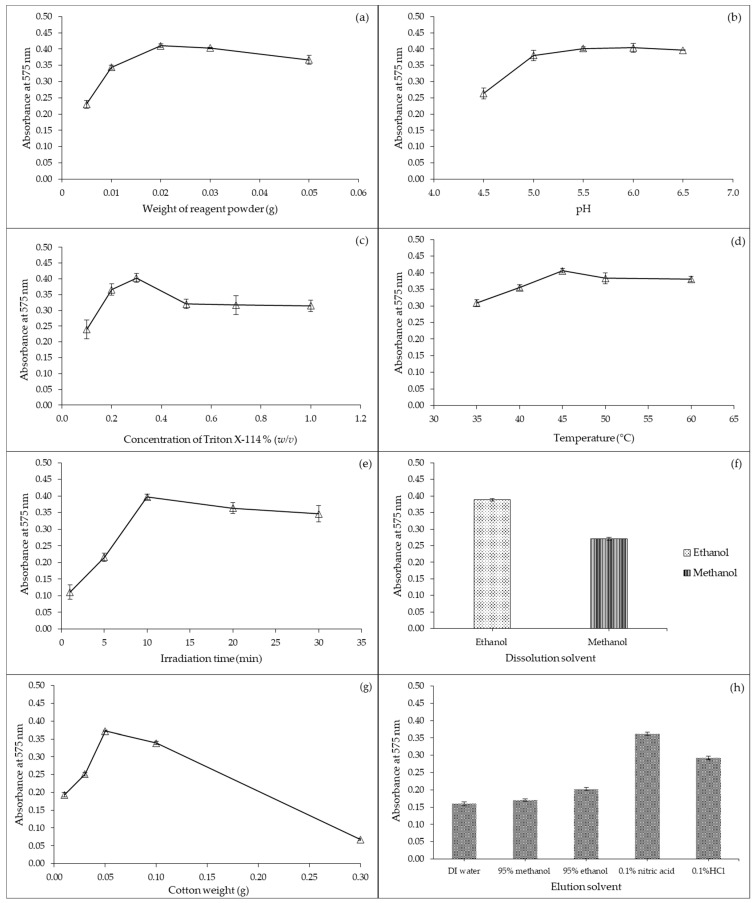
Optimal conditions of various parameters on sensitivity for the determination of total iron by the developed method at maximum wavelength 575 nm; (**a**) Effect of amount of *Dipterocarpus intricatus* Dyer fruit powder; (**b**) Effect of pH; (**c**) Effect of Triton X-114 concentration; (**d**) Effect of temperature; (**e**) Effect of ultrasonic irradiation; (**f**) Effect of dissolution solvent; (**g**) Effect of cotton weight and (**h**) Effect of elution solvent.

The effect of the amount of *Dipterocarpus intricatus* Dyer on the absorption value of the complex between iron(III) and the reagent was investigated at 0.005, 0.01, 0.02, 0.03, and 0.05 g under 5.0 mL of 1.0 mol·L^−1^ acetate buffer pH 5.0, 0.03 g Triton X-114, 1 min of vortex, incubation at 45 °C for 10 min, centrifugation at 5000 rpm for 10 min, 0.05 g of cotton, 1.0 mL of ethanol, and 1.0 mL of 0.1% nitric acid. The absorbance signal at 575 nm increased with an increasing amount of reagent powder from 0.005 to 0.02 g (Figure 4a), and then slightly decreased. An excessive amount of reagent powder, iron(III) ions, and/or iron(III)-reagent complex adsorbed on the reagent powder made it difficult to elute iron(III)-reagent complex by fixing the volume of elution solvent and resulted in a decreased signal. Moreover, the absorption value of the blank solution increased at 0.05 g. Therefore, the amount of reagent was selected at 0.02 g for the determination of iron by the developed method.

#### 2.2.2. Effect of pH

The effect of pH on the sensitivity of iron quantification was examined using acetate buffer in the range from 4.5 to 5.5 and phosphate buffer from pH 6.0 to 6.5 by fixing reagent powder at 0.02 g, 5.0 mL of 1.0 mol·L^−1^ buffer, 0.03 g Triton X-114, 1 min of vortex, incubation at 45 °C for 10 min, centrifugation at 5000 rpm for 10 min, 0.05 g of cotton, 1.0 mL of ethanol, and 1.0 mL of 0.1% nitric acid. The absorbance signal was augmented from pH 4.5 to 5.0, while above 5.0 the signal leveled off (Figure 4b). Solution pH also influenced the dissociation of hydroxyl groups present on active sites of the natural reagent, affecting the stability of the complex between metal ions and ligands [48]. Functional groups of the reagent were protonated at low pH. Hence, the pH of the reaction between iron(III) with natural reagent was operated at an acetate buffer pH 5.5.

#### 2.2.3. Effect of Buffer Concentration and Volume

Acetate buffer concentration was investigated in the range from 0.1 to 1.0 mol·L^−1^ under reagent powder at 0.02 g, 5.0 mL of acetate buffer pH 5.5, 0.03 g Triton X-114, 1 min of vortex, incubation at 45 °C for 10 min, centrifugation at 5000 rpm for 10 min, 0.05 g of cotton, 1.0 mL of ethanol, and 1.0 mL of 0.1% nitric acid. Absorbance increased with increasing buffer concentration from 0.1 to 1.0 mol·L^−1^. High buffer concentration adequately controlled the solution pH, while ionic strength also increased at high buffer concentration, with aggregation of the surfactant-rich phase from the aqueous phase giving increased phase separation [49]. 

The volume of acetate buffer was varied at 1.0, 3.0, 5.0, and 7.0 mL by fixing the reagent powder at 0.02 g, 1.0 mol·L^−1^ acetate buffer pH 5.5, 0.03 g Triton X-114, 1 min of vortex, incubation at 45 °C for 10 min, centrifugation at 5000 rpm for 10 min, 0.05 g of cotton, 1.0 mL of ethanol, and 1.0 mL of 0.1% nitric acid. Absorbance increased from 1.0 to 3.0 mL and then the signal did not change. Therefore, the concentration and volume of acetate buffer were selected at 1.0 mol·L^−1^ and 3.0 mL, respectively. Acetate buffer concentration and volume both affected the ability to control the pH of the reaction. Low concentration and small buffer volume provide low sensitivity to control the pH of the mixed solution. Excessive volume and/or high buffer concentration impacted the pH and enhanced the ionic strength of the system. Increasing the ionic strength of the surfactant solution enhanced phase separation [49]. Further salt addition was not required in this system.

#### 2.2.4. Effect of Triton X-114 Concentration

Triton X-114 is often used as the surfactant in cloud point extraction to preconcentrate the complexation product between iron(III) and the natural reagent to increase sensitivity with low cost, minimal hazard, high density, and low cloud point temperature [30]. Triton X-114 concentration was tested at 0.1, 0.2, 0.3, 0.5, 0.7, and 1.0% (*w*/*v*) using the reagent powder at 0.02 g, 3.0 mL of 1.0 mol·L^−1^ acetate buffer pH 5.5, 1 min of vortex, incubation at 45 °C for 10 min, centrifugation at 5000 rpm for 10 min, 0.05 g of cotton, 1.0 mL of ethanol, and 1.0 mL of 0.1% nitric acid. Results are shown in Figure 4c. The absorbance surged from 0.1 to 0.3% (*w/v*) and then the sensitivity declined due to dilution of the surfactant phase. Hence, concentration of Triton X-114 was optimized at 0.3% (*w*/*v*). Cloud point extraction offers high efficiency as a simple operation with rapid extraction involving small volumes of dilution solvent. Therefore, this method is inexpensive and friendly to both the operator and the environment [26,27,49].

#### 2.2.5. Effect of Mixing Period

To homogenize the solution mixture and increase extraction efficiency, vortexing was investigated at 30 s, and 1, 2, 3, and 5 min by employing 0.02 g of *Dipterocarpus intricatus* Dyer fruit powder, 3.0 mL of 1.0 mol·L^−1^ acetate buffer pH 5.5, 0.03 g Triton X-114, incubation at 45 °C for 10 min, centrifugation at 5000 rpm for 10 min, 0.05 g of cotton, 1.0 mL of ethanol, and 1.0 mL of 0.1% nitric acid. Results of absorbance obtained by varying the vortex time were not different (data not shown). Therefore, vortexing for 30 s was adopted for the proposed method to reduce analysis time.

#### 2.2.6. Effect of Temperature

Triton X-114 clouds at room temperature (25 °C) and increasing temperature above the cloud point of the surfactant increased extraction efficiency. Cloud point temperature was examined between 35 and 60 °C by fixing 0.02 g of natural reagent powder, 3.0 mL of 1.0 mol·L^−1^ acetate buffer pH 5.5, 0.03 g Triton X-114, 30 s of vortex, incubation of 10 min, centrifugation at 5000 rpm for 10 min, 0.05 g of cotton, 1.0 mL of ethanol, and 1.0 mL of 0.1% nitric acid. Absorption value was highest when incubating the mixed solution at 45 °C. For incubation temperatures at 50 and 60 °C, the absorbance decreased slightly because of the unstable reaction complex (Figure 4d). Therefore, extraction of reagent, complexation, and preconcentration were conducted at 45 °C.

#### 2.2.7. Effect of Ultrasonic Irradiation

The effect of ultrasonic irradiation time was studied from 1 to 30 min under 0.02 g of natural reagent powder, 3.0 mL of 1.0 mol·L^−1^ acetate buffer pH 5.5, 0.03 g Triton X-114, 30 s of vortex, incubation at 45 °C, centrifugation at 5000 rpm for 10 min, 0.05 g of cotton, 1.0 mL of ethanol, and 1.0 mL of 0.1% nitric acid. Results are presented in Figure 4e. The absorbance signal surged with increasing irradiation time from 1 to 10 min, while at over 10 min sensitivity decreased because the surfactant micellar aggregates broke down and dissolved into the aqueous phase [50]. Hence, the ultrasonic incubation time was optimized at 10 min. Ultrasonic radiation was utilized to increase both reagent extraction and cloud point extraction. During reagent extraction, ultrasonic irradiation enhanced the release of phenolic compounds from *Dipterocarpus intricatus* Dyer fruit powder into the liquid phase through the acoustic cavitation phenomena and chemical effects [51,52]. Ultrasound irradiation accelerated the mass transfer of the target analyte from the aqueous phase into the extracting micelle mediator [50,53]. 

#### 2.2.8. Centrifugation Speed and Time

Centrifugation speed to aggregate phase separation was examined between 3000 and 6000 rpm by employing 0.02 g of natural reagent powder, 3.0 mL of 1.0 mol·L^−1^ acetate buffer pH 5.5, 0.03 g Triton X-114, 30 s of vortex, incubate at 45 °C for 10 min, centrifugation for 10 min, 0.05 g of cotton, 1.0 mL of ethanol, and 1.0 mL of 0.1% nitric acid. The signal achieved from different speeds was not different. Therefore, centrifugation speed was selected at 5000 rpm. Centrifuge time was tested from 5 to 30 min. Absorbance values were not different. Hence, 10 min of centrifugation was chosen.

#### 2.2.9. Study of the Dissolution Solvent

Ethanol and methanol were tested as organic solvents to dissolve the extraction phase to reduce the viscosity (experimental conditions: 0.02 g of natural reagent powder, 3.0 mL of 1.0 mol·L^−1^ acetate buffer pH 5.5, 0.03 g Triton X-114, 30 s of vortex, incubation at 45 °C for 10 min, centrifugation at 5000 rpm for 10 min, 0.05 g of cotton, and 1.0 mL of 0.1% nitric acid). The surfactant-rich phase remained at the bottom of the centrifuge tube and was not appropriate for measuring the absorbance by a spectrophotometer due to its high viscosity. Using ethanol to dissolve the surfactant phase provided the highest absorbance, as presented in Figure 4f. Therefore, ethanol was selected to dissolve surfactant in the developed method because this solvent achieved high sensitivity and less toxicity than methanol. 

Volumes ranging from 0.1 to 2.0 mL were investigated by fixing reagent powder at 0.02 g, 3.0 mL of 1.0 mol·L^−1^ acetate buffer pH 5.5, 0.03 g Triton X-114, 30 s of vortex, incubation at 45 °C for 10 min, centrifugation at 5000 rpm for 10 min, 0.05 g of cotton, ethanol as dissolution solvent, and 1.0 mL of 0.1% nitric acid. Absorbance increased at a volume of ethanol from 0.1 to 1.0 mL and then decreased above 1.0 mL due to the dilution effect. Thus, 1.0 mL of ethanol was selected to dilute the surfactant.

#### 2.2.10. Study of the Filtering Process

Weight of cotton was varied in the range from 0.01 to 0.3 g under conditions of 0.02 g of natural reagent powder, 3.0 mL of 1.0 mol·L^−1^ acetate buffer pH 5.5, 0.03 g Triton X-114, 30 s of vortex, incubation at 45 °C for 10 min, centrifugation at 5000 rpm for 10 min, 1.0 mL of ethanol, and 1.0 mL of 0.1% nitric acid. Cotton packing in the 3 mL syringe barrel was used to filter and trap the reagent powders. At between 0.01 and 0.03 g, the cotton was not sufficient to filter and trap the reagent powders, resulting in a turbid solution that was unsuitable to measure absorbance. Weight of cotton that was more than 0.05 g caused absorbance to decrease (Figure 4g) because the reaction product was absorbed by the cotton and was, thus, difficult to eluate with elution solvent. Therefore, 0.05 g of cotton was selected as appropriate.

#### 2.2.11. Study of the Elution Solvent

Proper eluent to elute the collected complexes from cotton was adapted from a previous study [54]. Different types of eluents as deionized water, 95% ethanol, 0.1% (*v/v*) nitric acid, and 0.1% (*v/v*) hydrochloric acid were used to elute the iron(III)-natural reagent complex adsorbing on the cotton by employing the other parameters at 0.02 g of natural reagent powder, 3.0 mL of 1.0 mol·L^−1^ acetate buffer pH 5.5, 0.03 g Triton X-114, 30 s of vortex, incubation at 45 °C for 10 min, centrifugation at 5000 rpm for 10 min, 0.05 g of cotton, 1.0 mL of ethanol, and 1.0 mL of elution solvent. Results (Figure 4h) showed that 0.1% nitric acid released the complex in the cotton and obtained the highest absorbance. Therefore, 0.1% nitric acid was adopted as the elution solvent.

Nitric acid concentration was investigated at 0.05, 0.1, 0.5, 1, 3, and 5% under the optimal conditions of 0.02 g of natural reagent powder, 3.0 mL of 1.0 mol·L^−1^ acetate buffer pH 5.5, 0.03 g Triton X-114, 30 s of vortex, incubation at 45 °C for 10 min, centrifugation at 5000 rpm for 10 min, 0.05 g of cotton, 1.0 mL of ethanol, and 0.1% nitric acid. Absorbance increased slightly with increasing nitric acid concentration from 0.05 to 0.1%, and then the signal declined above 0.1% due to the change in solution pH. Thus, 0.1% nitric acid was selected. Volume of 0.1% nitric acid was studied between 1.0 and 3.0 mL. Highest absorbance was obtained at 1.5 mL of 0.1% nitric acid. Dilution of the solution was observed when the volume of 0.1% nitric acid was more than 1.5 mL, yielding a reduced absorption value. Hence, 1.5 mL of 0.1% nitric acid was employed for the developed method.

### 2.3. Analytical Performance of the Proposed Method

Method validation as linearity range, limit of detection (LOD), limit of quantification (LOQ), and precision represented in terms of relative standard deviation (RSD) were examined under the optimal/selected conditions for the determination of iron. A calibration graph for iron determination was obtained in the range from 0.1 to 1.0 mg·L^−1^ with *r^2^* more than 0.997. LOD and LOQ were calculated by 3SD/S and 10SD/S, where SD is the standard deviation of the blank (*n* = 7) and S is the slope of the calibration graph at 0.03 and 0.09 mg·L^−1^. RSD values of the proposed method tested at 0.1 and 0.5 mg·L^−1^ of iron(III) were 1.5% and 0.7%, respectively for intra-day (*n* = 10) and 2.3% and 0.9% for inter-day (*n* = 10 × 5), indicating that this method provided good precision. To prevent experimental inherent variability using the natural reagent, the calibration curve for the determination of iron by the proposed method should be constructed daily, with absorbance measured against the blank solution. Analytical figures of merit for the proposed method were compared with other natural reagents in Table 1. The proposed method provided high sensitivity as a green alternative analytical method based on natural reagent extract from *Dipterocarpus intricatus* Dyer to determine iron.

### 2.4. Interference Study

Some metal elements and anions interfered in the analysis of iron by our proposed method. This may lead to incorrect concentrations of iron in real samples. The interferences were investigated by increasing the concentration of the interferent with the analyte of interest at 0.5 mg·L^−1^. Tolerance limits, defined as the interferent concentration, caused an error of absorbance ±5% at 0.5 mg·L^−1^ of iron(III). The ions Pb^2+^, Cr(VI), Ni^2+^, Cd^2+^, Hg^2+^, and Cd^2+^ also interfered at 10 mg·L^−1^. Tolerance limits of Al^3+^ and Cu^2+^ were 15 and 20 mg·L^−1^, respectively while Na^+^, Ca^2+^, Mg^2+^, K^+^, and Cl^−^ interfered at 50 mg·L^−1^ and Zn^2+^, and Mn^2+^ interfered for iron assay at 100 mg·L^−1^. Anions such as CO_3_^2-^ and NO_3_^−^ provided tolerance limits at 200 mg·L^−1^. The tolerance concentration of iron(II) was 5 mg·L^−1^.

### 2.5. Application to Local Vegetable Samples

Our developed method quantified iron in green vegetable samples. Local vegetables such as Chinese kale, sweet basil, *Tiliacora triandra* leaf, and Siamese neem flower were selected as representative samples to study the accuracy of this method by spiking standard iron at 0.5 mg·L^−1^. Recoveries ranged from 89.0 to 99.8%, indicating that the proposed method provided high accuracy. Our method was also applied to assay iron content in eight vegetables. Iron was detected in all samples between 2.45 and 13.36 mg/100 g, as shown in Table 2. Results agreed with FAAS evaluated by F-test and *t*-test at 95% confidence level (F_calculate_ = 1.106, F_critical_ = 3.787 and *t_calculate_* = 0.297, *t_critical_* = 2.364 at df = 7). This green alternative method to quantify iron concentration in vegetable samples was cost-effective, reliable, and convenient, with high precision, accuracy, and environmentally friendly.

## 3. Materials and Methods

### 3.1. Chemical and Reagents

All chemicals and reagents used were analytical reagent grade, with solutions prepared by dissolution with deionized water (Milli Q, Millipore, Germany).

A standard stock solution of 1000 mg·L^−1^ iron(III) for atomic absorption spectrometry (AAS) was obtained from Merck, Germany. A working solution of iron(III) standard in the range from 0.1 to 1.0 mg·L^−1^ was generated by diluting 1000 mg·L^−1^ iron(III) with deionized water.

Stock solution of Mn^2+^, Mo(VI), Pb^2+^, Cr(VI), Ni^2+^, Cd^2+^, Hg^2+^, Cu^2+^, Al^3+^, Ca^2+^, Mg^2+^, K^+^ and Na^+^ (1000 mg·L^−1^) for AAS was purchased from Merck, Germany. Fe^2+^ stock solution at 1000 mg·L^−1^ was prepared from iron(II) sulfate heptahydrate (Merck, Germany).

Acetate buffer pH 5.5 at 1.0 mol·L^−1^ was prepared by weighing 110 g of sodium acetate trihydrate (Ajax Finechem, Auckland, New Zealand) and then mixing with 8.45 mL of 98% acetic acid (Merck, Darmstadt, Germany). The final pH was adjusted using 1.0 mol·L^−1^ sodium hydroxide (Ajax Finechem, Auckland, New Zealand) and the volume was made up to 1000 mL with deionized water.

Triton X-114 non-ionic surfactant (Sigma-Aldrich, Darmstadt, Germany) was employed to preconcentrate the reaction product between iron(III) and the natural chelating agent by cloud point extraction (CPE) before spectrophotometric analysis at 575 nm. 

Tannic acid standard, nitric acid, and 95% ethanol were purchased from Merck (Merck, Darmstadt, Germany).

### 3.2. Instruments

UV-Visible spectroscopy (UV-1800, Shimadzu, Kyoto, Japan) was employed to scan spectra and measure the absorption values of the extraction phases. FT-IR (INVENIO-S, Bruker, Leipzig, Germany) was used to confirm the functional groups of the natural reagent extracted from *Dipterocarpus intricatus* Dyer and compare results with the tannic acid standard. An ultrasonic bath (Elmasonic S 30 H, Elma, Singen, Germany) was utilized to assist the extraction. A benchtop centrifuge (Hettich Zentrifugen, Tuttlingen, Germany) was used to separate the surfactant-rich phase, and a cooking blender (Electrolux, Bangkok, Thailand) was employed to grind the natural reagent and vegetable samples. A furnace (CWF, Carbolite, Hope, UK) was utilized to incinerate vegetable samples. All pH measurements were made using a 713-pH meter (Metrohm, Herisau, Switzerland). A flame atomic absorption spectrometer (FAAS, Agilent Technologies, Santa Clara, CA, USA) was used to detect iron concentration as the standard method, with results compared to the developed method.

### 3.3. Dipterocarpus intricatus Dyer Fruit Reagent Powder Preparation

*Dipterocarpus intricatus* Dyer is a local plant found in Thailand. The fruit was harvested between February and May from Rio-Et and Maha Sarakham Provinces. The dry fruit was cleaned with tissue to remove the dirt and then desiccated in an oven at 60 °C for 24 h before grinding to powder using a cooking blender. The reagent powder was packed in polyethylene zip bags and kept at room temperature in a desiccator.

### 3.4. Ultrasound-Assisted One-Pot Cloud Point Extraction for Iron Determination

**Figure 5:**
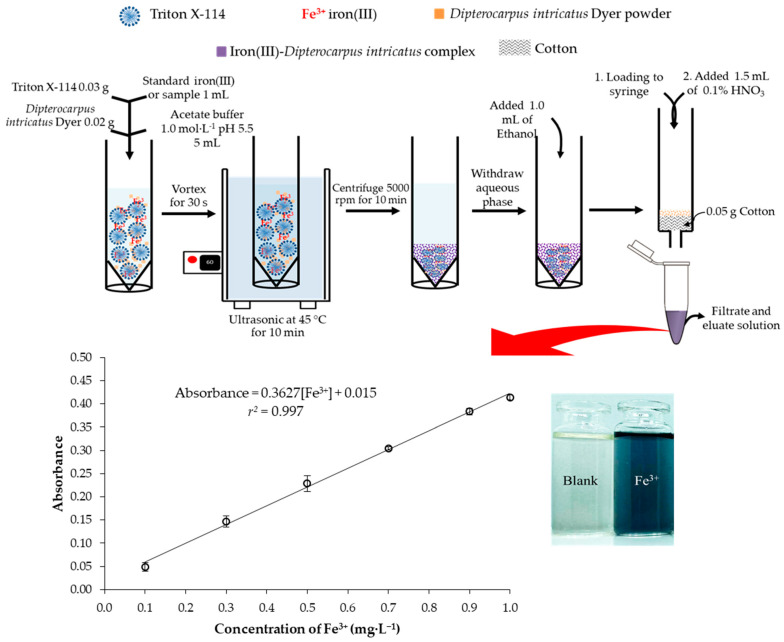
Ultrasound-assisted one-pot operational steps to determine iron by the developed method.

Determination of iron by the proposed method followed a one-pot reaction involving natural reagent extraction, complexation reaction of iron between the natural chelating agent, and cloud point preconcentration. Ultrasonication was utilized to assist and increase extraction efficiency. The determination steps involved weighing *Dipterocarpus intricatus* Dyer powder 0.02 g into a 15 mL centrifuge tube. Then, 0.03 g of Triton X-114 was added. Various volumes between 0.1 and 1.0 mL of 10 mg·L^−1^ iron(III) standard solution (final concentration in 10 mL of iron ranged from 0.1 to 1.0 mg·L^−1^) or 1.0 mL of sample solution were transferred into a centrifuge tube. After that, 3.0 mL of 1.0 mol·L^−1^ of acetate buffer pH 5.5 was added to control the pH of the solution. The volume was then adjusted to 10 mL with deionized water and vortexed for 30 s before the solution was transferred into an ultrasonic bath at 45 °C for 10 min (37 kHz of ultrasonic frequency and 80 W of ultrasonic power effective) for simultaneous one-pot cloud point extraction including natural reagent extraction, complexation, and preconcentration. The dark-blue cloudy solution was cooled to room temperature and then centrifuged at 5000 rpm for 10 min. The surfactant-rich phase was obtained and the upper supernatant solution was withdrawn using a long-tubed needle before dilution with 1.0 mL ethanol 95% to decrease the viscosity. The solution was then passed through a syringe barrel containing 0.05 g cotton to filter and trap the *Dipterocarpus intricatus* Dyer powder. Reaction products adsorbed on the cotton were eluted by 1.5 mL of 0.1% nitric acid. The mixed solution (filtrate and eluate) was measured for absorbance at 575 nm by a spectrophotometer. The operational steps are illustrated in Figure 5.

### 3.5. Vegetable Samples

Green vegetable samples including Chinese kale, sweet basil, *Tiliacora triandra* leaf, Siamese neem flower, wildbetal leafbush, Thai copper pod, peppermint leaf, and Turkey berry fruit were purchased from a local market in Maha Sarakham Province, Thailand. Sample preparation was adopted and modified from previous procedures for iron determination [22,55,56]. Briefly, the samples were washed with deionized water and then desiccated at 60 °C in an oven for 24 h. The dried samples were ground using a cooking blender and accurately weighed at 1.00 g into a crucible before adding 0.5 mg·L^−1^ iron standard for recovery study. Then, 1.0 mL of 65% nitric acid was added and the samples were incinerated at 450 °C for 16 h in a furnace. Each dry ash sample was redigested with 1.0 mL of concentrated nitric acid and incinerated at 450 °C for 6 h. Next, the residual dry ash sample was added with 1.0 mL of concentrated nitric acid and 5.0 mL of deionized water. The solution was filtered through filter paper into a 25 mL volumetric flask, and the volume was adjusted to 25 mL with deionized water. The samples were diluted with deionized water before analysis by the proposed method to obtain a final iron concentration within the calibration range.

### 3.6. Standard FAAS Method

The FAAS method was employed for quantification of total iron concentration in real samples as the standard method to compare results with our proposed method. The FAAS conditions were set as airflow 13.50 L min^−1^, acetylene flow 2.00 L min^−1^, burner height 13.5 mm, burner length 10 cm, and detection wavelength 248.3 nm. A calibration graph was plotted for concentrations ranging from 0.2-10.0 mg·L^−1^ to determine the total iron contents in the samples.

## 4. Conclusions

Green analytical chemistry using *Dipterocarpus intricatus* Dyer fruit as a natural chelating agent for the determination of total iron was developed. Ultrasonic radiation was used to assist a one-pot reaction comprising reagent extraction and preconcentration based on cloud point to enhance sensitivity. This developed method was successfully applied to quantify iron content in vegetable samples. Results of iron concentration by the proposed method were not significantly different from those achieved using the FAAS standard method. Our proposed method using ultrasound-assisted one-pot cloud point extraction with natural reagent from *Dipterocarpus intricatus* Dyer fruit was cost-effective, reliable, eco-friendly, and convenient as an alternative green analytical approach for the determination of iron contents in vegetable samples.

## Figures and Tables

**Table 1:** Analytical results of figures of merit for the proposed method compared with other natural reagents.

Natural Reagent.	Analytical Method	Preconcentration Method	Sample	Linearity (mg·L^−^^1^)	LOD/LOQ (mg·L^−^^1^)	%RSD	Ref.
Indian gooseberry	Flow injection spectrophotometry	-	Pharmaceutical preparations and water samples, groundwater, and tap water	0.5–20.0	0.31/0.5	2.02–2.32	[19]
Guava leaves	Flow injection spectrophotometry	-	Tap water	1.0–10.0	1/-	3–10	[20]
Green tea	Flow injection spectrophotometry	-	Pharmaceutical preparations	1.0–20.0	0.05/-	1.1–7.1	[21]
Betel nut	Sequential injection spectrophotometry	-	Rice	0.2–10	0.06/0.20	<5	[22]
Lead tree	Spectrophotometry	-	Blood tonic	0–10	0.2/0.7	<5	[24]
*Smilax china* root	Sequential injection spectrophotometry	-	Groundwater	1.0–8.0	0.05/0.17	2.6	[25]
*Dipterocarpus intricatus* Dyer	Spectrophotometry	Cloud point extraction	Vegetable	0.1–1.0	0.03/0.09	0.9–2.3	This study

**Table 2:** Iron concentration in vegetables analyzed by the developed method and FAAS.

Vegetable Sample	Iron (mg/100 g ±SD, *n* = 3)	
	Proposed	FAAS
Chinese kale	3.47 ± 0.20	3.51 ± 0.30
Sweet basil	2.45 ± 0.30	2.48 ± 0.24
*Tiliacora triandra* leaf	8.76 ± 0.29	9.34 ± 0.39
Siamese neem flower	4.49 ± 0.19	4.68 ± 0.20
Wildbetal leafbush	4.94 ± 0.10	5.32 ± 0.26
Thai copper pod	13.36 ± 0.95	12.44 ± 0.94
Peppermint leaf	8.87 ± 0.43	9.20 ± 0.26
Turkey berry fruit	5.25 ± 0.27	5.01 ± 0.15

## Data Availability

Not applicable.
